# The Role and Efficacy of Coenzyme Q10 in the Management of Erectile Dysfunction in a Hypertensive Male: An Interventional Study

**DOI:** 10.7759/cureus.17937

**Published:** 2021-09-13

**Authors:** Victor H Aguilera-Alvarez, Bilal Khan Mohammed, Aqsa Fatima, Ankit Patel, Avaniben Patel, Frederick N Gyabaah, Jobby John, Abbas Iqbal, Sidra Naz, Affan Munir, Ammer Haffar, Muhammad Irfan, Muhammad Hanif

**Affiliations:** 1 Health Policy, Imperial College London, London, GBR; 2 Internal Medicine, Deccan College of Medical Sciences, Hyderabad, IND; 3 Internal Medicine, Allama Iqbal Medical College, Lahore, PAK; 4 Internal Medicine, Spartan Health Science University School of Medicine, St. Lucia, USA; 5 Clinical Research, Texas Center for Drug Development, Texas, USA; 6 Internal Medicine, Dr. Somervell Memorial CSI Medical College and Hospital, Kerala, IND; 7 Pediatrics, Ayub Teaching Hospital, Abbottabad, PAK; 8 Internal Medicine, University of Health Sciences (UHS), Lahore, PAK; 9 Internal Medicine, Jersey City Medical Center, Jersey City, USA; 10 Internal Medicine, Hayatabad Medical Complex, Peshawar, PAK; 11 Internal Medicine, Khyber Medical College Peshawar, Hayatabad Medical Complex, Peshawar, PAK

**Keywords:** b-blocker, erectile dysfunction, coenzyme q10, alpha blocker, hypertension

## Abstract

Introduction

Erectile dysfunction (ED) is a prevalent medical condition that affects millions of men globally. A number of pharmacological and complementary options are used in the management of ED, including Coenzyme Q10 (CoQ10). Oxidative stress has been linked to the progression of ED, and Co Q10 protects against oxidative damages and improves erectile function as well as the activity of antioxidant enzymes. This study aimed to determine the efficacy of CoQ10 in the treatment of erectile dysfunction in hypertensive males.

Method

An open-labeled parallel arm interventional study was conducted in the cardiology unit of Hayatabad Medical Complex Hospital, Peshawar, Pakistan, from March 2020 to March 2021. Hypertensive male patients (n = 230) were randomly allocated to either receiving 200-gram CoQ10 daily along with their current antihypertensive therapy (n=104) or anti‐hypertensive treatment only (n=105). The patient’s erectile function was assessed at baseline and three months using the International Index of Erectile Function Test (IIEF-5) during the study period.

Result

Of the total 230, 209 (90.87%) patients were included in the final analysis. There were no significant differences in demographics, history of illness, co-morbid conditions, and current medication of both groups. After three months, 21 (20.1%) participants scored more than 17 in the IIEF-5 and no longer had ED. Overall, no significant difference was found in the mean IIEF-5 score between the study group and control group (14.41 ± 4.49 Vs. 15.61 ± 4.82; p=0.06). However, in subgroup analysis, significant improvement in the study group was seen in participants with mild ED (p=0.03).

Conclusion

With the demonstration of its efficacy in hypertensive patients with mild ED, co-enzyme Q10 supplementation can be proposed as a potential candidate in patients with long-term hypertension and can play a role in erectile dysfunction.

## Introduction

Inability or failure of erection during sexual intercourse is termed impotence or erectile dysfunction(ED) [1]. The prevalence widely varies across countries. The prevalence of ED ranges from 3-76.5% globally [2]. ED is significantly linked to age as its prevalence increases from 5% at age 40 to 15% at 70 years. Age is the most important factor in impotence; other risk factors include heart diseases, diabetes mellitus, hypertension, psychological, certain drugs, and smoking [3-4]. The prevalence of ED has been shown to be 58.3% in hypertensive patients, considerably high than the general population. About 21%of hypertensive males had severe ED, with 20.7 % and 16.4 % reporting moderate and mild ED, respectively [5]. Several studies have also observed a notable relationship between hypertension and ED [2,4-5].

The most prevalent comorbidity among ED patients is hypertension. This condition has an impact on the patients' and their wives' or partners' quality of life [6-7]. Sexual dysfunction is a common problem among hypertensive individuals, and it may occur as a side effect of various antihypertensive drugs [4-5,7]. Antihypertensive drugs of various types can decrease blood pressure in a similar way, but they have different effects on ED [7]. According to Lundberg and Biriell [8] and Doumas et al. [9], alpha-blocking or alpha/beta-blocking drugs and guanidine derivatives are more likely to cause ED than calcium-blocking drugs, converting enzyme inhibitors, or diuretics. Burchardt et al. described that patients with concomitant ED and hypertension had a higher risk of cardiovascular issues [10].

A number of pharmacological and complementary options are used in the management of ED, including CoQ10 [11]. CoQ10 has antioxidant properties and protects against oxidative damages occurring in the body. Many clinical trials, systematic reviews, and much of the research demonstrated the use and benefits of CoQ10 as a supplement to treat various diseases [12]. The progression of ED has been linked to oxidative stress, thus CoQ10 improves erectile function by protecting against oxidative damage. After collecting data from preclinical and clinical studies, most of the results have clearly shown that CoQ10 is highly safe for use as a dietary supplement [13].

Although the promising results of CoQ10 supplementation in erectile dysfunction are seen in few studies, still there is very limited and contradictory data. Therefore, further studies to evaluate and determine the efficacy of CoQ10 in the treatment of impotence in hypertensive males is the need of the hour.

## Materials and methods

An open-labeled, parallel-arm, interventional study was conducted in the cardiology unit of a tertiary care hospital in Pakistan from March 2020 to March 2021. A total of 230 (n=230) male hypertensive patients, with the age range of 41 to 70 years, with complaints of ED for at least the last three months, were enrolled in this study. The sample size was calculated using Gpower software® to consider an effect size of 0.36, a power of 80%, and a 5% level of significance. The study sample was selected via the consecutive non-probability sampling method, and written informed consent was taken. The institutional ethical review board approved the study protocol (Ref No=176/HEC/B&PC/21) prior to data collection. Patients on beta-blockers were not included in the study, as it has an impact on erectile function. Participants were randomized via online software “Research Randomizer” (https://www.randomizer.org/) at an allocation ratio of 1:1 into two groups. The study group was randomized to receive 200-gram CoQ10 daily along with their current antihypertensive therapy (n=104), and the control group received no additional treatment to their anti‐hypertensive treatment (n=105).

After enrollment, detailed medical history was taken to record all comorbidities and medication on a self-structured questionnaire. The patient’s erectile function was assessed using the International Index of Erectile Function Test (IIEF-5) [14-15]. The IIEF score ranged from 0 to 25, with the lowest score being 0 and the highest being 25. An IIEF-5 score below 21 was considered ED. According to the classification by the IIEF-5 test, scores ranging from 5 to 7 were considered severe, 8-11 were moderate, 12-16 were mild-moderate, 17-21 were mild, whereas scores above 21 were considered normal [16]. The principal investigator conducted the interview after explaining the purpose of the study. IIEF-5 questions were explained to participants in their native language. Privacy of patients was ensured as interviews were taken in separate rooms. Patients were asked to return for follow-up after every month. At the end of three months, IIEF-5 was repeated. A total of 21 patients were lost to follow-up; 11 from the study group and 10 patients from the control group. Only those participants who completed the study were included in the final analysis.

Statistical analysis was done using the Statistical Package for the Social Sciences (SPSS v. 23.0) (IBM Corp., Armonk, New York). Numerical data were presented as mean and standard deviation while categorical data were presented as frequency and percentages. Between the groups, mean differences were calculated using the independent t-test, and the association between categorical variables was determined using the chi-square test. Mean values at day 0 and day 90 for the study and control group were compared using a dependent t-test. A p-value of ≤0.05 was considered statistically significant.

## Results

The mean age of patients in the study group was 51.0 ± 11.0 years, whereas the control group was 52.0 ± 13.0 years and there was no significant difference. The proportion of smokers in both groups was almost similar. Similarly, there were no significant differences in co-morbid conditions, history of life-threatening events, such as stroke or heart attack, and medication history between the two groups (Table 1).

**Table 1:** Demographics of the enrolled participants and their medications for hypertension ACEI: angiotensin-converting enzyme inhibitor, ARB: angiotensin receptor blocker, NS: non-significant

Characteristics	Study Group (n=104)	Control Group (n=105)	p-value
Age (Mean ± SD)	51.0 ± 11.0	52.0 ± 13.0	NS
Smokers	51 (49.0%)	49 (46.7%)	NS
Comorbidity			
Diabetes mellitus	34 (32.7%)	38 (36.2%)	NS
Chronic liver diseases	06 (5.8%)	06 (5.7%)	NS
Chronic kidney diseases	11 (10.6%)	10 (9.5%)	NS
Hyperlipidemia	41 (39.4%)	43 (41.0%)	NS
Asthma	05 (4.8%)	06 (5.7%)	NS
History of stroke	04 (3.8%)	04 (3.8%)	NS
History of myocardial infarction	06 (5.8%)	05 (4.8%)	NS
Benign prostatic hyperplasia	19 (18.3%)	16 (15.2%)	
Medications for hypertension			
Alpha-blockers	05 (4.8%)	04 (3.8%)	NS
ACEi/ARBs	91 (87.5%)	93 (88.6%)	NS
Calcium channel blockers	26 (25.0%)	30 (28.6%)	NS
Diuretics	27 (26.0%)	28 (26.7%)	NS

In the study group after three months, 21 (20.1%) participants reported scores more than 17 in IIEF-5 and were considered normal. There was no significant difference in the mean IIEF-5 in the study group and control group (14.41 ± 4.49 vs. 15.61 ± 4.82; p=0.06). On subgroup analysis, significant improvement was seen in participants with mild ED scores (p=0.03) (Table 2).

**Table 2:** Comparison of scores based on IIEF-5 on day 0 and day 90 of both groups ED: erectile dysfunction, IIEF-5: International Index of Erectile Function Test

IIEF-5	Study Group (n=104)			Control Group (n=105)		
	Day 0	Day 90*	p-value	Day 0	Day 90	p-value
Mild ED (17-21)	33 (31.7%)	20 (19.2%)	0.03	33 (31.4%)	31 (29.5%)	0.76
Mild-Moderate ED (12-16)	30 (28.8%)	26 (25.0%)	0.53	28 (26.6%)	29 (27.6%)	0.87
Moderate ED (8-11)	31 (29.8%)	28 (26.9%)	0.64	33 (31.4%)	32 (30.4%)	0.75
Severe ED (5-7)	10 (9.6%)	9 (8.6%)	0.80	11 (10.47%)	12 (11.4%)	0.82
IIEF-5Score (mean±SD)	14.41 ± 4.49	15.61 ± 4.82	0.06	13.79 ± 5.01	13.90 ± 5.12	0.87

## Discussion

The most prevalent comorbidity among ED patients is hypertension, and more than half of the hypertensive males present with ED as a complication of hypertension [17]. Although it can be managed with the addition of more drugs to the preexisting list of medications, one proposed intervention is supplementation with CoQ10. Our results showed that the use of 200g CoQ10 daily, for a period of three months, improved the status of mild ED in hypertensive males, with 20.1% of patients reporting complete resolution. However, the same efficacy was not noticed in hypertensive males with moderate to severe ED.

The prevalence of ED in long-term hypertension (>5 years) can be explained by the high levels of angiotensin II in these patients. It acts on angiotensin II type 1 receptor to promote vasoconstriction of corporal smooth muscles, induce vascular hypertrophy and endothelial dysfunction via the activation of nicotinamide adenine dinucleotide phosphate (NADPH) resulting in the generation of reactive oxygen species (ROS), which deplete nitric oxide (NO) required for vasodilation, necessary during sexual excitation for erection [18-19]. These actions lead to the modulation of tone in the penile arteries and the vascular smooth muscles, resulting in erectile dysfunction.

CoQ10 (ubiquinone) is a mitochondrial coenzyme that undergoes oxidation-reduction reactions to facilitate ATP production. It is found to have antioxidant properties due to the high concentration of quinol in its membranes, which function by either directly neutralizing free radicals or by replenishing other antioxidants like vitamin C (ascorbate) and E (tocopherol) [20].

Kedziora-Kornatowska et al. reported that the supplementation of CoQ10 results in enhancing the availability of NO by scavenging superoxides and plays a role in promoting endothelium-dependent vasodilation in patients with hypertension [21]. This increases the blood flow and can help in explaining its beneficial effect in patients with ED.

The use of CoQ10 has not been studied in hypertensive males with ED before, but there are several studies regarding its use in other diseases exploring the same principle of action. In a study conducted on men with Peyronie's disease, supplementation with 300mg of CoQ10 daily showed improvement in ED [12]. Zozina et al. stated that cardiac myocyte hypertrophy is promoted by oxidative stress and cytokines, and the use of CoQ10 has been shown to reduce this by hindering the initial process of ROS production [22]. Ju et al. in their study stated that an important pathogenetic factor for retinal neurodegeneration is oxidative stress-mediated mitochondrial dysfunction. The use of CoQ10 in such patients has shown promising results and has been proposed as a therapeutic agent to halt ischemic retinal neurodegeneration [23]. Ho et al. conducted clinical trials on hypertensive patients with the supplementation of CoQ10 and demonstrated a mean reduction of 11 mmHg in systolic blood pressure and 7 mmHg in diastolic blood pressure, however, they concluded that it is still uncertain whether CoQ10 helps in blood pressure reduction in long term hypertension [24]. This may help explain the role of CoQ10 in improving ED.

Perhaps, this is the first study that investigates the role of CoQ10 in ED in hypertensive patients. However, since it was a single-center study, care should be taking while inferring the result to a larger population. Further, large-scale studies are needed to determine the efficacy and dose of CoQ10 in patients suffering from erectile dysfunction in hypertensive patients.

## Conclusions

In conclusion, the supplementation of CoQ10 in hypertensive males with mild ED shows improvement as compared to the control group, which only received the regular antihypertensive medication. The proposed mechanism is its antioxidant properties and vasodilation effect. The lack of response in moderate to severe ED necessitates further investigation as one of the factors may be an inadequate time of observation and dosage as compared to the severity of ED.

## References

[1] El Senoussi A, Coleman DR, Tauber AS (1959). Factors in male impotence. J Psychol.

[2] Kessler A, Sollie S, Challacombe B, Briggs K, Van Hemelrijck M (2019). The global prevalence of erectile dysfunction: a review. BJU Int.

[3] Feldman HA, Goldstein I, Hatzichristou DG, Krane RJ, McKinlay JB (1994). Impotence and its medical and psychosocial correlates: results of the Massachusetts Male Aging Study. J Urol.

[4] Bruzziches R, Francomano D, Gareri P, Lenzi A, Aversa A (2013). An update on pharmacological treatment of erectile dysfunction with phosphodiesterase type 5 inhibitors. Expert Opin Pharmacother.

[5] Bener A, Al-Ansari A, Afifi M, Krishna PV (2007). Erectile dysfunction among hypertensive men in a rapidly developing country. Indian J Urol.

[6] Kushiro T, Takahashi A, Saito F (2005). Erectile dysfunction and its influence on quality of life in patients with essential hypertension. Am J Hypertens.

[7] Mittawae B, El-Nashaar AR, Fouda A, Magdy M, Shamloul R (2006). Incidence of erectile dysfunction in 800 hypertensive patients: a multicenter Egyptian national study. Urology.

[8] Lundberg P, Biriell C (1993). Impotence—the drug risk factor. Int J Impot Res.

[9] Doumas M, Tsakiris A, Douma S (2006). Beneficial effects of switching from beta-blockers to nebivolol on the erectile function of hypertensive patients. Asian J Androl.

[10] Burchardt M, Burchardt T, Baer L (2000). Hypertension is associated with severe erectile dysfunction. J Urol.

[11] Hernández-Camacho JD, Bernier M, López-Lluch G, Navas P (2018). Coenzyme Q10 supplementation in aging and disease. Front Physiol.

[12] Safarinejad MR (2010). Safety and efficacy of coenzyme Q10 supplementation in early chronic Peyronie's disease: a double-blind, placebo-controlled randomized study. Int J Impot Res.

[13] Hidaka T, Fujii K, Funahashi I, Fukutomi N, Hosoe K (2008). Safety assessment of coenzyme Q10 (CoQ10). Biofactors.

[14] Rosen RC, Riley A, Wagner G, Osterloh IH, Kirkpatrick J, Mishra A (1997). The international index of erectile function (IIEF): a multidimensional scale for assessment of erectile dysfunction. Urology.

[15] Cappelleri JC, Rosen RC, Smith MD, Mishra A, Osterloh IH (1999). Diagnostic evaluation of the erectile function domain of the international index of erectile function. Urology.

[16] Rosen RC, Cappelleri JC, Gendrano N 3rd (2002). The International Index of Erectile Function (IIEF): a state-of-the-science review. Int J Impot Res.

[17] Kloner R (2007). Erectile dysfunction and hypertension. Int J Impot Res.

[18] Nunes KP, Labazi H, Webb RC (2012). New insights into hypertension-associated erectile dysfunction. Curr Opin Nephrol Hypertens.

[19] Doumas M, Douma S (2006). Sexual dysfunction in essential hypertension: myth or reality?. J Clin Hypertens (Greenwich).

[20] Crane FL (2001). Biochemical functions of coenzyme Q10. J Am Coll Nutr.

[21] Kędziora-Kornatowska K, Czuczejko J, Motyl J (2010). Effects of coenzyme Q10 supplementation on activities of selected antioxidative enzymes and lipid peroxidation in hypertensive patients treated with indapamide. A pilot study. Arch Med Sci.

[22] Zozina VI, Covantev S, Goroshko OA, Krasnykh LM, Kukes VG (2018). Coenzyme Q10 in cardiovascular and metabolic diseases: current state of the problem. Curr Cardiol Rev.

[23] Martucci A, Nucci C (2019). Evidence on neuroprotective properties of coenzyme Q10 in the treatment of glaucoma. Neural Regen Res.

[24] Ho MJ, Li EC, Wright JM (2016). Blood pressure lowering efficacy of coenzyme Q10 for primary hypertension. Cochrane Database Syst Rev.

